# Traumatic spinal injuries in the Kingdom of Saudi Arabia: a study of associated injuries, management and mortality

**DOI:** 10.11604/pamj.2019.32.153.18047

**Published:** 2019-04-01

**Authors:** Khalid Mansour Alkhathlan, Mohammad Ghormallah Alzahrani, Khalid Hadi Aldosari, Mohammed Ibrahim Alsheddi, Abdullah Abdulrahman Alqeair

**Affiliations:** 1 Prince Sattam Bin Abdulaziz University, Colleges of Medicine, Al-Kharj, Kingdom of Saudi Arabia; 2 Almaarefauniversity, College of Medicine and Surgery, Riyadh, Kingdom of Saudi Arabia

**Keywords:** Traumatic, spinal, injuries

## Abstract

**Introduction:**

traumatic spinal fracture is a painful and disabling injury associated with poor long-term functional outcome. The objective of the present study was to assess the frequency of spinal fractures in road traffic accident (RTA) victims, their management, mortality rate and associated injuries. This study reveals and adds useful insights to the literature from Kingdom of Saudi Arabia (KSA) in terms of incidence of RTA-related spinal fractures, including their management and mortality rate.

**Methods:**

a cross-sectional study was conducted at King Khalid Hospital and Prince Sultan Center for Health Services (KKH & PSCHS) in AlKharj, KSA from September 2016 to June 2017. A total of 120 patients suffering from spinal/vertebral fractures due to RTAs were included in this study. The data was collected from patients' charts, including age, gender, region or distribution of the spinal fracture, associated fractures, number of fractures, degrees of shock, admission to intensive care unit (ICU), treatment modalities, along with the management of spinal fractures, days of hospital stay, referral and discharges or deaths.

**Results:**

the mean age of patients was 29.21. The most common anatomic region of the fracture was the cervical region (35%). Injuries associated with traumatic spinal fracture were predominated by clavicular fractures. More than half of the victims (58.30%) had a cervical brace applied before leaving the hospital. 29.20% patients required posterior stabilization with pedicle screws. Anterior corpectomy, grafting and plating was done to 4.30% patients.

**Conclusion:**

traumatic spinal fractures require prompt diagnosis and timely management in order to improve the outcome.

## Introduction

A spinal fracture is a disabling condition that imposes a significant effect on the patient's quality of life. It may potentially lead to disability, inability to work and poor social and financial outcomes. Road traffic accidents (RTA) are considered the most frequent cause of spinal fractures all over the world [1]. The reason RTAs are the most common cause of spinal fractures may be attributed to the increasing number of vehicles on the roads. According to one estimate, KSA will have 10.03 million vehicles by 2020 [2]. RTAs are estimated to account for between 20.9% and 33.6% of all spinal fractures [3]. Generally, spinal fractures affect young adults and indicate a higher degree of injury severity, in terms of associated injuries and increased mortality rate [1, 4]. RTAs result in a plethora of traumatic spinal injuries and associated injuries, depending on certain factors such as age, speed of the vehicle, type of crash and type of vehicle. Motor vehicle accidents account for 80.1% of spinal injuries in KSA [5]. Among RTAs, car accidents are a major cause of spinal fracture, contributing to 40% of RTA-related spinal injuries, where the most common mechanism of spinal injury is car-rollover [6, 7]. RTA-related vertebral fractures pose a higher severity of trauma, morbidity and mortality rate [5]. Increased severity, morbidity and mortality of patients with RTA-related spinal fractures depend upon several factors, such as old age, associated life-threatening injuries or penetrating injuries, and co-morbid conditions [8]. Unfortunately, spinal injuries (6.6%) are often missed during primary survey at the accident site, which results in increased disability, and risk of death [9]. The reasons spinal fractures are missed may include lack of obvious spinal deformity or radiological findings. In these circumstances, spinal stabilization is a key intervention to avoid further damage to the patient. Therefore, when dealing with victims of RTAs, it is necessary to carefully examine the spine at the accident site, during the transportation of the victims and in the hospital. Common injuries, which accompany spinal or vertebral fractures, include injuries to extremities, head and chest, including fractures of ribs and the sternum [10]. A spinal fracture with concomitant rib or sternal fractures will adversely affect the outcome, and may cause neurological dysfunction. Studies have reported rib fractures in 7.2% to 17.8% of patients suffering from traumatic spinal fractures [10]. This means that patients with injuries to extremities, head, neck and chest are at higher risk of accompanied spinal trauma. Hence, it is critical to look for a spinal injury and ensure stabilization to avoid further harming the patient. Delaying the diagnosis and management of spinal fractures increases morbidity and mortality [10].

The thoracolumbar region is the most common site involved in spinal or vertebral fractures [11]. Freitas *et al.* conducted a prospective study on vertebral fractures in 5995 elderly patients (age > 65 years) suffering from traumatic spinal fractures, and reported that 33.3% of vertebral fractures occurred at the thoracic level, 56% of fractures in the lumbar region and 10.7% of fractures in the cervical region [11]. In another cross-sectional study, Wang *et al.* [10] studied 698 patients with traumatic fractures in order to determine the incidence and pattern of spinal fractures in patients involved in motor vehicle accidents in China. They reported most common vertebral fractures at L1 (19.2%) and T12 vertebra (11.3%), followed by fractures at C2 level (8.3%). However, they reported that the most common site of spinal fractures among motorcyclists was at C3-C7. The reason motorcyclists sustain more spinal injuries may be that they are thrown from their bikes and are not protected by car compartments. In KSA, a retrospective study that included 1428 patients with 2056 fractures was conducted to determine the pattern of traumatic fractures and dislocations. The authors reported maximum fractures in extremities following 323 spinal fractures. Road traffic accidents were the major cause of fractures. However, the study did not mention the regions of the spine involved in the fractures. Therefore, the present study is a valuable addition to the medical literature on spinal fractures in KSA. The management of spinal fractures includes pre-hospital care, emergency department care and surgical therapy. Pre-hospital management emphasizes the safe transportation of patients with spinal fractures to the emergency department, without causing further harm. At this stage, spinal immobilization is of prime importance. In the emergency department, the protocol of basic life support should be followed, along with a secondary survey. The most important management at this stage is to stabilize the spine, keeping the spinal collar in place and treating the spinal shock with emergency medical therapy. Patients with spinal fractures and neurological deficits should be treated with high-dose steroids. Spinal fractures can be managed conservatively or operatively. Minor fractures with a stable vertebral column are treated conservatively using a spinal orthotic vest or brace and intravenous steroids if indicated [12]. On the other hand, spinal fractures with vertebral column instability are managed operatively. The purpose of surgical therapy (i.e. posterior stabilization with pedicle screws/brace/skeletal traction, anterior corpectomy, grafting and plating) is to decompress the spinal cord canal, and stabilize the vertebral column [12]. An RTA-related spinal fracture is a painful and disabling injury associated with poor long-term functional outcome (6%) [13]. The present study assesses the frequency of spinal fractures in RTA victims, their management, mortality rate and associated injuries. Comprehensive literature on the incidence of RTA-related spinal fractures in the Kingdom of Saudi Arabia (KSA) is lacking, including patient management and mortality rate. Hence, the present study is an important addition to the study.

## Methods

We conducted a retrospective study, which included 120 patients suffering from spinal/vertebral fractures due to RTAs, at King Khalid Hospital and Prince Sultan Center for Health Services (KKH & PSCHS) in AlKharj, Kingdom of Saudi Arabia (KSA) for 9 months from September 2016 to June 2017. Situated in Al Kharj Industrial City, KKH & PSCHS has adopted a National Healthcare Delivery System to cope with the rising needs of a vast surrounding population. KKH & PSCHS has served the community for the past 36 years, and is dedicated to providing excellent healthcare services, patient safety and health education in the region. The purpose of this study was to determine frequency, mortality rate, associated injuries and their management in patients with spinal fractures due to an RTA. The study was initiated after having obtained consent from the Ethical Review Committee of the hospital. The data was collected from the files and charts of patients who were admitted and treated for spinal fractures due to an RTA. Patients who had a clear diagnosis of a spinal fracture due to an RTA, confirmed by radiological imaging, were included in the study. Patients were excluded from the study if they had incomplete data, no radiological evidence to confirm the injury, had left the hospital against medical advice (LAMA), or were suspected to being involved in criminal activity. The data, collected from the patients' charts, included age, gender, region or distribution of spinal fracture, associated fractures, number of fractures, degrees of shock, admission to ICU, all the modalities of treatment along with the management of the spinal fractures, days of hospital stay, referral and discharges or deaths. The hospital was visited on a daily basis to collect and record data on a proforma (attached) that was specially designed for the study. The data was saved in softcopy as well as in hard copies in respective file folders, and proper coding was done. The data was properly managed by retaining the completed proforma and entering the data on a daily basis in SPSS-21 for statistical analysis. Data cleaning was done to check for any missing data or improperly filled questionnaires during data collection. Quantitative variables, such as age, number of fractures and duration of accident at presentation were derived by calculating the mean and standard deviations. Qualitative variables such as gender, marital status, ethnicity, education and mortality were derived by calculating frequency and percentages. After data analysis, the results were presented in the form of tables, graphs and figures.

## Results

A total of 120 patients were included in the study.

**Demographic results:** the mean age of patients was 29.21, with a standard deviation of 10.472, and 50 (41.7%) of them were young adults (21-30 years old). Out of 120 patients, 87.5% of patients were male, and 12.5% female. Males predominated (87.5%) with a male to female ratio of 7:1. The socio-demographic characteristics are shown in Table 1.

**Table 1:** Demographic breakdown of the data

Age category	Frequency	Percentage
0-20	15	12.5 %
21-30	50	41.7 %
31-40	35	29.2 %
41-50	10	8.3 %
51-60	10	8.3 %
**Mortality**		
Dead	15	12.5 %
Alive	105	87.5 %
**Gender**		
Female	15	12.5%
Male	105	87.5%
**Shock Degree**		
First degree	25	20.8%
Second degree	20	16.7%
Third degree	5	4.2%
Fourth degree	0	0.0%
No shock	70	58.3%
**Operation**		
Yes	55	45.8%
No	65	54.2%
**ICU admission**		
Yes	40	33.3%
No	80	66.7%
**Referred to another hospital**		
Yes	55	45.8%
No	65	54.2%

**Figure 1:**
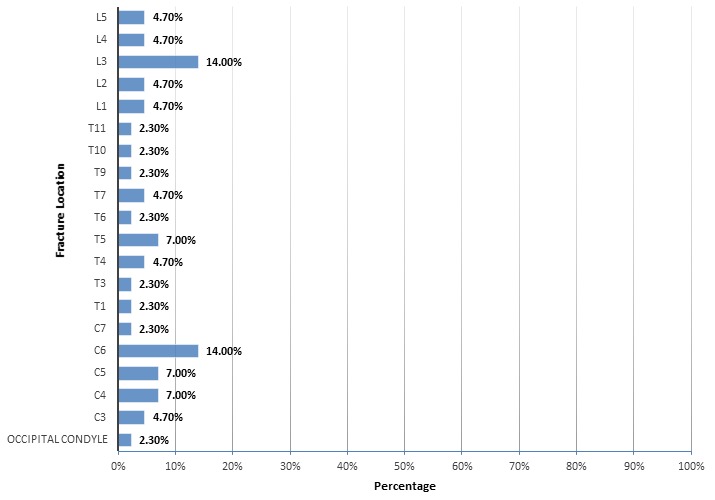
Spinal fracture distribution

**Anatomical sites of fractures:** the most common anatomic region of the fractures was the cervical region (35%), followed by the lumbar region (32.8%) and then the thoracic region (30.2%). Some of the patients (3.6%) developed occipital condyle fractures. Overall, the cervical spine was the most commonly injured spinal segment. The most commonly injured vertebral levels were C6 (14%) and L3 (14%). The highest neurologic level of injury was C3, while the lowest was L5. (Figure 1)

**Figure 2:**
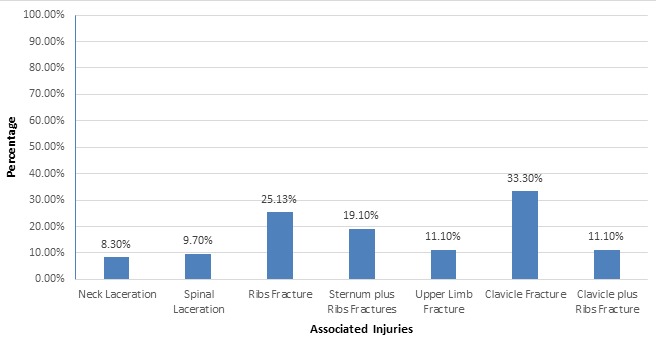
Associated injuries with traumatic spinal fracture

**Associated injuries:** injuries associated with a traumatic spinal fracture were predominated by clavicular fractures (33.30%), followed by single or multiple rib fractures (25.13%). A combined fracture of the ribs and sternum occurred in 19.10% of patients. Upper limb fractures were present in 11.10% of the patients. Both the clavicle and ribs were fractured in 11.10% patients. Spinal lacerations were present in 9.70% of the cases. An associated neck laceration was also reported in 8.30% of patients. (Figure 2)

**Figure 3:**
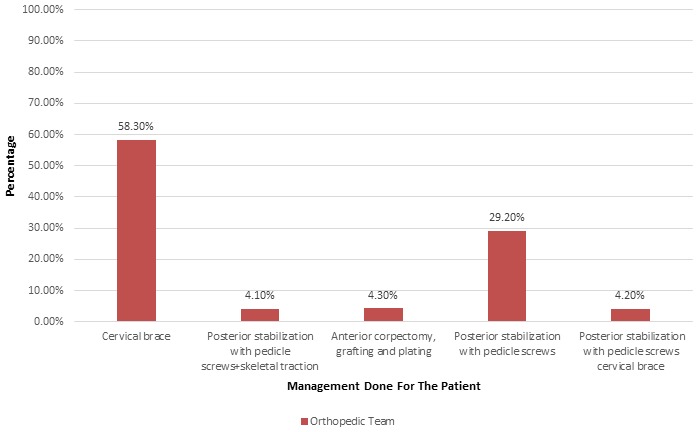
Orthopedic management of patients with a traumatic spinal fracture

**Figure 4:**
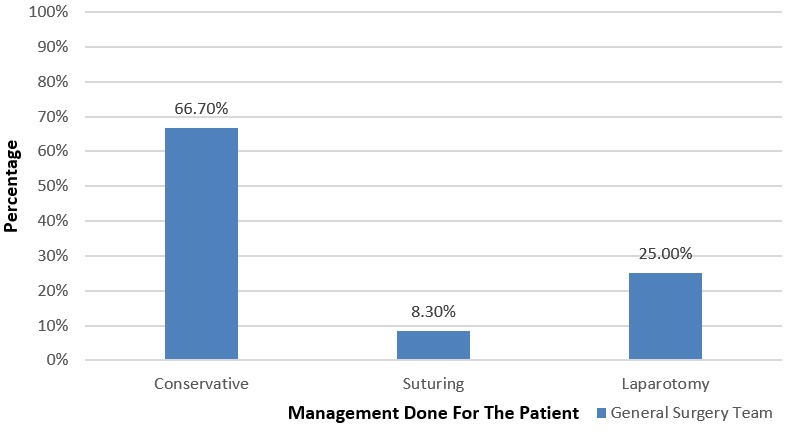
Surgical management of patients with a traumatic spinal fracture

**Management:** more than half of the victims (58.30%) had applied a cervical brace before leaving the hospital. Posterior stabilization with pedicle screws was performed on 29.20% of patients. Anterior corpectomy, grafting and plating was done in 4.30% of patients. Posterior stabilization with pedicle screws and cervical brace was done in 4.20% of patients. Similarly, posterior stabilization with pedicle screws and skeletal traction was done in 4.10% of patients (Figure 3). Overall, 55 (45.8%) victims were operated on, out of which conservative surgery (66.70%) predominated, followed by laparotomy (25%) and suturing (8.30%) (Figure 4).

**Degrees of shock:** more than half (58.3%) of the patients did not develop shock, and were stable. Of those remaining, 20.8% developed first degree shock, 16.7% developed second degree shock, and only 4.2% were reported to have developed third degree shock. 33.3% were admitted to the ICU from the accident and emergency department, and 48.8% of victims were referred to another hospital. There was an overall mortality rate of 12.5% (Table 1).

## Discussion

Spinal fracture, a painful and disabling condition, significantly affects a patient's quality of life in terms inability to work and poor socioeconomic outcome. In this retrospective study, the majority of the patients were young adult males. The reason behind the predominance of male gender is the jurisdiction in the country, wherein, the women were not allowed to drive until June 2018 [14]. The most common site of spinal fractures was the cervical region, followed by the lumbar and thoracic regions, with a few patients having an occipital condyle fracture. The most commonly injured single vertebra was C6, followed by L3. The highest neurologic level of injury was C3, while the lowest was L5. Clavicle and rib fractures were the most common types of injuries associated with spinal fractures. Management of spinal fractures included conservative surgery, laparotomy and suturing. About one-third of the patients required ICU admission, and a few patients developed third degree shock. Mortality was reported in 12.5% cases. The most common victims of spinal or vertebral fractures are adolescents and young adults. Ovalle *et al.* [15] conducted a retrospective cross-sectional study comprising of 60 patients to assess the incidence and functional outcomes of traumatic spinal injuries. The authors reported an average age of 35 years, with injuries to the thoracic region being the most common. In another study, most common traumatic spinal injuries and complications were reported in the age group of 20-39 years [10]. In the present study, the mean age of the victims was reported as 29.21 years. In this regard, the present study is supported by the literature, in terms of age and spinal injuries. Traumatic fracture levels are important in terms of patient management and functional outcome. These levels of spinal fracture vary in the literature. Wang *et al.* [8] retrospectively reviewed 698 patients with traumatic spinal fractures at Third Military Medical University-affiliated hospitals located in Southwest China. They reported that the most common traumatic fracture was at L1 (19.2%), followed by T12 (11.3%) and C2 (8.3%). In this context, the results of the present study do not match the results of research by Wang *et al* [10]. In their study, the most common victims were car drivers. However, they reported that the most common fractures among motorcycle drivers were at C3-C7. The reason behind the higher rate of cervical injuries in motorcycle riders may be attributed to being thrown from their bikes without being protected by car compartments. This means that the type of vehicle involved in MVAs affects the level of spinal fractures. Similarly, contrary to the results of the present study, Freitas *et al.* [11] reported that the majority of injuries were in the thoracolumbar region. This anomaly may be explained by their inclusion of traumatic spinal injuries other than those caused by RTAs.

However, there is a need for a large study to determine the relationship between the type of motor vehicle and level of traumatic spinal fractures. Similarly, Ovalle *et al.* [15] reported that the most common traumatic spinal injuries occurred in the thoracolumbar region. Yunoki *et al.* [16] retrospectively reviewed 134 patients with traumatic intracranial hemorrhage (ICH), skull fractures and spinal fractures among those whowere injured in traffic accidents or falls. They reported spinal fractures in 10 patients, with the thoracolumbar region being the most common location of spinal fractures. In contrast, the most common level of spinal fractures seen in the present study was at C6, followed byL3. This difference between these two studies may be due to a number of factors, including the type of vehicle [10], type of trauma [11], vehicle speed at the time of the accident and preventive measures, such as seat belts. Spinal fractures are often accompanied by associated injuries, adding to the severity, morbidity and mortality. Therefore, it is critical to identify associated injuries in the patients with traumatic spinal fractures, as these significantly affect the outcome. Wang *et al.* [10] reported associated injuries in 38.4% patients, including thoracic (44.4%), head and neck (25%), extremities (13.8%), pelvic (8.6%) and abdominal (1.9%) injuries associated with traumatic spinal injuries. In the present study, clavicle and rib fractures were the most common types of injuries associated with traumatic spinal fractures. Other associated injuries included upper extremity fractures, spinal neck lacerations and fractures of the sternum. Management of traumatic spinal fractures is again a critical element, as missing or delaying the identification of spinal fractures leads to worse functional outcomes. Crucially, it is critical to identify and care for any spinal fracture at the accident site, during the transportation, and in the hospital. Usually, management of traumatic spinal fractures includes stabilization of the spinal column, decompression of spinal nerves, and surgery [17]. The most important management at this stage is to stabilize the spine, keeping the spinal collar in place and treating the spinal shock with emergency medical therapy. In the present study, patients with traumatic spinal fractures were managed with cervical collar, cervical brace, anterior or posterior stabilization with pedicle screws, anterior corpectomy, grafting and plating and skeletal traction. Traumatic spinal fractures are associated with higher severity, morbidity and mortality [5]. Serious spinal fractures (especially those with associated injuries) most often require ICU admission for intensive care. Such patients may require blood transfusions, particularly those who present with second and third degree shock. However, in the present study, the degree of shock did not affect the mortality rate. The present study offers reliable local data related to traumatic spinal injuries, associated injuries and mortality, which may provoke interest in conducting further studies in order to validate the results of the present study and to implement policies to prevent spinal injuries at the local level. However, single-centered retrospective design of the present study does not enable the generalization of the study. Also, the present study had not determined the relationship of associated injuries in terms of mortality. Therefore, multi-centered prospective studies are required to validate the results of the present study.

## Conclusion

In conclusion, a traumatic spinal fracture is a disabling condition which affects functional outcome. As a rule of thumb, prompt diagnosis and timely proper management improves the outcome. Moreover, the implementation of traffic laws regarding speed limits, drinking, helmet and seatbelt usage will reduce MVAs and traumatic spinal fractures in KSA.

### What is known about this topic

- Road traffic accidents (RTA) are considered the most frequent cause of spinal fractures all over the world;
- Spinal injuries (6.6%) are often missed during primary survey at the accident site, which results in increased disability, and risk of death;
- Common injuries, which accompany spinal or vertebral fractures, include injuries to extremities, head and chest, including fractures of ribs and the sternum.

### What this study adds

- The most common anatomic region of the fractures was the cervical region;
- Injuries associated with a traumatic spinal fracture were predominated by clavicular fractures (33.30%);
- The present study offers reliable local data related to traumatic spinal injuries, associated injuries and mortality, which may provoke interest in conducting further studies in order to validate the results of the present study and to implement policies to prevent spinal injuries at the local level.

## Competing interests

The authors declare no competing interests.
